# Flat Die Extruded Biocompatible Poly(Lactic Acid) (PLA)/Poly(Butylene Succinate) (PBS) Based Films

**DOI:** 10.3390/polym11111857

**Published:** 2019-11-11

**Authors:** Vito Gigante, Maria-Beatrice Coltelli, Alessandro Vannozzi, Luca Panariello, Alessandra Fusco, Luisa Trombi, Giovanna Donnarumma, Serena Danti, Andrea Lazzeri

**Affiliations:** 1 Department of Civil and Industrial Engineering, University of Pisa, 56122 Pisa, Italy; vito.gigante@dici.unipi.it (V.G.); alessandrovannozzi91@hotmail.it (A.V.); luca.panariello@ing.unipi.it (L.P.); serena.danti@unipi.it (S.D.); andrea.lazzeri@unipi.it (A.L.); 2 Consorzio Interuniversitario Nazionale per la Scienza e Tecnologia dei Materiali (INSTM), 50121 Florence, Italy; alessandra.fusco@unicampania.it (A.F.); l.trombi@yahoo.it (L.T.); giovanna.donnarumma@unicampania.it (G.D.); 3 Department of Experimental Medicine, University of Campania “Luigi Vanvitelli”, 80138 Naples, Italy; 4 OTOLAB, Azienda Ospedaliero-Universitaria Pisana (AOUP), 56122 Pisa, Italy

**Keywords:** poly(lactic acid), poly(butylene succinate), flat die extrusion, biocompatibility

## Abstract

Biodegradable polymers are promising materials for films and sheets used in many widely diffused applications like packaging, personal care products and sanitary products, where the synergy of high biocompatibility and reduced environmental impact can be particularly significant. Plasticized poly(lactic acid) (PLA)/poly(butylene succinate) (PBS) blend-based films, showing high cytocompatibility and improved flexibility than pure PLA, were prepared by laboratory extrusion and their processability was controlled by the use of a few percent of a commercial melt strength enhancer, based on acrylic copolymers and micro-calcium carbonate. The melt strength enhancer was also found effective in reducing the crystallinity of the films. The process was upscaled by producing flat die extruded films in which elongation at break and tear resistance were improved than pure PLA. The in vitro biocompatibility, investigated through the contact of flat die extruded films with cells, namely, keratinocytes and mesenchymal stromal cells, resulted improved with respect to low density polyethylene (LDPE). Moreover, the PLA-based materials were able to affect immunomodulatory behavior of cells and showed a slight indirect anti-microbial effect. These properties could be exploited in several applications, where the contact with skin and body is relevant.

## 1. Introduction

About 150 million tons of plastic per year are consumed worldwide, which are often disposed of in landfills, where degradation rate is tremendously slow [1]. The development of new materials, derived from renewable resources, must challenge high technologicaland environmental compliance [2]. By replacing the commodity fossil-based polymers with biodegradable polymers, which are readily susceptible to microbial action [3], more sustainable waste management of post-consumer plastics can be favored.

Biodegradable polymers have also risen the interest to produce films and sheets, in applications like packaging and skin contact items, often used in sanitary and personal care products. Both the research and the industry sectors are focusing on the development of new, environment–friendly materials, which are obtained from alternative and renewable resources, with reduced energy consumption and possibly without negative impacts on the environment [4]. So far, biodegradable polymers are more expensive than the traditional thermoplastic counterparts; furthermore, they show properties which are still unsatisfactory to many practical applications, such as mechanical and barrier properties. It is, therefore, necessary to improve such properties in order to make them competitive towards the fossil-based thermoplastic polymers [5,6].

Poly(lactic acid) (PLA) is probably the most popular fully renewable and biodegradable polymer used in various applications, due to its high tensile strength, good heat stability during processing, excellent biocompatibility and excellent gloss and clarity. However, important drawbacks, including brittleness and poor heat resistance, have been reported [7]. To overcome these defects and make the PLA useful even in appliances in which high flexibility and toughness are necessary, several studies on PLA blends with other biodegradable polymers, such as with polycaprolactone [8,9], poly(butylene succinate) (PBS) [10,11,12], polybutylene(succinate-co-adipate) [13,14], and poly(butylene adipate-co-terephthalate) (PBAT) [15,16,17,18,19], were investigated. Among these, a fully biobased and biodegradable solution is obtained by blending PLA with PBS, that is a biodegradable aliphatic polyester produced by the polycondensation reaction of 1, 4-butanediol with succinic acid [20,21,22,23,24]. PBS has high flexibility, excellent impact strength, as well as thermal and chemical resistance. Moreover, many studies on PBS, in the form of films and molded objects, have exhibited significant biodegradation within several months in soil, water with activated sludge, and seawater [25,26].

Plastic films are highly used in products to be in contact with human bodies, such as food packaging, personal care and sanitary products. Films and multilayer systems can be manufactured using different converting processes, such as blown film extrusion, flat die extrusion, extrusion coating, extrusion laminating and co-extrusion, depending on the material type in use, the width and thickness of the film and the required film properties [27]. In this study, flat die extrusion, operating through a linear die of an adjustable thickness (i.e., die gap) usually between 3.0 mm and 1.4 mm, has been used to produce polymeric sheets and films with thicknesses ranging from 50 microns to 1 mm. The geometry generally used for the flat die distribution channel in the extruder head is the coat-hanger die, equipped with a straight distributor that ensures better homogeneity in the flow. Due to the small size of the supply section, heavy head loss occurs in the head, and the pressure can reach up to 300 bar, locally. Hence, the flow control rods must be provided with gaskets to prevent the escaping of melt polymer [28]. The material that comes out from the die in the form of a molten plate goes immediately in contact with a system of thermostatic rollers to allow cooling and solidification. Then, formulating blends with the suitable rheological properties for modulating final flexibility is fundamental.

To obtain flexible PLA-based blends, maximizing the biobased content of the material, the addition of suitable biobased plasticizers is considered fundamental [29]. Indeed, in the last decade, many studies were carried out about PLA blends with other biodegradable polyesters and plasticizers prepared by flat die extrusion. For example, plasticized and nano-filled films were produced and tested by Scatto et al. [30], by using o-acetyl tri-n-butyl citrate (ATBC) as plasticizer. The rheological measurements performed by capillary viscometer suggested that the processability of plasticized and nanofilled PLA is suitable for the industrial production of cast films. These authors stated that plasticization allowed to obtain a material ductile at room temperature. Good miscibility of the plasticizer is, thus, important to avoid de-mixing and loss in the transparency of films [31]. Moreover, the plasticizer should not migrate out of the film [32].

The use of chain extenders or peroxides can also be crucial for modulating the rheological properties increasing melt viscosity by introducing some branching, and hence, a shear thinning behavior [33]. Moreover, additives with antimicrobial properties (e.g., chitin nanofibrils) are usually added to these film formulations [34,35]. Market formulations of these films are often very complex, as they contain fillers, such as calcium carbonate and talc, or nucleating agents, to control the crystallization of PLA during processing by increasing resistance without compromising the transparency. A patent to produce transparent PLA-based blends containing organic plasticizers with epoxy functions and biodegradable elastomeric polyesters, which allows the production of transparent PLA-based films, maintaining compostability, has been reported [36,37,38]. In the class of citrates, both renewable and biodegradable, ATBC can be considered a good candidate for obtaining plasticized flat die extruded flexible films made of PLA/PBS blends [39,40,41,42,43,44,45].

Usually, melt strengtheners, commercially available, are used to increase the viscosity and the melt strength of plasticized blends to allow the production of films, thus, allowing good control of processability and final properties [46,47,48,49]. In particular, Hernandez et al. [50] stated that PLA needs to be melt-strengthened to enlarge its processing window and range of applications; therefore, different chain extenders have been used, mainly based on epoxy groups reactivity of styrenic, acrylic and glycidyl acrylic copolymers [51], or acrylic copolymers. Recently, it was found that acrylic copolymers resulted quite efficient for increasing melt viscosity in PLA/PBAT plasticized blends. As PLA-based blends have shown to be reactive towards radical reactions [52], probably a similar mechanism can also be hypothesized during the reactive extrusion of biopolyesters with this melt strength enhancer [53]. However, other authors highlighted that the increase in melt viscosity could be explained by the occurrence of not negligible interactions between PLA and acrylic polymer [54] or copolymers [55], associated to their good miscibility with PLA. Nevertheless, the mechanism explaining melt strength enhancement has not fully been clarified up to now.

Antiblock (also called “antitack”) additives are commonly employed to improve the processing of polymer films. More specifically, this class of additives is able to reduce the adhesion between films in the winding phase [56]. Antiblock agents, typically finely divided solid minerals, such as natural silica, talc and calcium carbonate, act by producing a slight roughening of the surface [57]. CaCO_3_ is one of the most common raw materials in nature, assumed to be about 4% of the Earth’s crust. Moreover, it meets the criteria of renewability as a biobased material, because it is continuously replenished through natural cycles in rivers, lakes and oceans or is formed as minerals in shells, skeletons, stalactites and stalagmites. Commercial grades of calcium carbonate are produced from natural sources, including limestone, chalk or marble with an annual replenishment of calcium carbonate of 8.8–14.5 billion tons/year in different environments. Therefore, according to the ISO 14021 definition, the replenishment rate exceeds the consumption rate [58,59].

In the present paper, films based on PLA/PBS blends with ATBC, a liquid plasticizer from renewable sources, were produced with and without CaCO_3,_ acting as slip and antiblocking agent, and acrylic copolymers, as melt strength enhancers at high shear rate. A preliminary study was performed by mini-extrusion trials followed by thermo-mechanical characterization, about the effect of the different additives. The films were then produced through a COMAC semi-industrial twin-screw extruder connected to a flat die and calendering system. On these samples, thermal, mechanical and in vitro biocompatibility properties, using both keratinocytes and mesenchymal stromal cells (hMSCs), to mimic human skin and connective tissues, respectively, have been analyzed, aiming to obtain flexible films with properties improved with respect to pure PLA, and biocompatibility improved with respect to common polyolefins.

## 2. Materials and Methods 

### 2.1. Materials

Poly(lactic acid) (PLA) Ingeo ™ 2003D, Extrusion Grade with a density of 1.24 g/cm^3^, produced by NatureWorks LLC was the commercial product used in this work. It is made up of 4.1% isomeric D units. The poly(Butylene succinate) (PBS) used in this work is BioPBS FD92PM with a density of 1.24 g/cm^3^, produced by Mitsubishi Chemical Corporation. It consists of a copolymer of succinic acid, adipic acid and 1,4-butandiol.

Acetyl tri-n-butylcitrate (ATBC), purchased from Tecnosintesi (Bergamo, Italy), is a liquid organic compound prepared by acetylation of tri-n-butylcitrate. It is bio-based and consists of a colorless liquid soluble in organic solvents. It has a density of 1.05 g/cm^3^ and a molecular weight of 402.5 g/mol. 

Ground CaCO_3_ (indicated as 2AV) was purchased from OMYA (Avenza, Carrara, Italy) under the trade name of OMYACARB 2-AV, manufactured from high purity and crystalline white marble. These CaCO_3_ particles are dry ground products that have a mean particle size (d 50%) of 2.6 μm, top cut diameter (d 98%) of 15 μm, cubic shape (aspect ratio = 1), and density of 2.7 g/cm^3^

Plastistrength 550 (indicated as PS for brevity) is a product of Arkema (Colombes Cedex, France). It is a medium molecular-weight acrylic copolymer. It is a commercial processing aid [60]. It appears as a white powder with a density of 1.17 g/cm^3^

Low density polyethylene (LDPE) reference is a product of Versalis S.p.A. (San Donato Milanese, Milan, Italy) under the name of RIBLENE FL 30. It is a low density polyethylene suitable to be transformed with blown film technology. Riblene FL 30 is, therefore, recommended for the extrusion of films for general uses, low thickness films, films for lamination and for blends. The suggested melt temperature range is from 160 to 190 °C.

HMSCs were supplied from Merck Millipore S.A.S., (Burlington, MA, USA). HaCaT, Dulbecco’s Minimal Essential Medium (DMEM), L-glutamine, penicillin, streptomycin and fetal calf serum were purchased from Invitrogen, (Carlsbad, CA, USA). Fetal Bovine Serum and AlamarBlue^®^ were purchased from Thermo Fisher Scientific, (Waltham, MA, USA). Dulbecco’s phosphate-buffered saline (DPBS) and magnesium chloride (MgCl_2_) were purchased from Sigma-Aldrich (Milan, Italy). Hexamer primers were bought from Promega (Madison, WI, USA). LC Fast Start DNA Master SYBR Green kit was obtained from Roche Applied Science (Penzberg, Germany). 

### 2.2. Methods

#### 2.2.1. Preparation of Blends

PLA/PBS blends were prepared by adding ATBC to make the product more flexible, CaCO_3_ acting as slip and antiblocking agent and PS acrylic copolymer for modulating the viscosity during processing (Table 1). 

The micro-compounder used to obtain rheological data is the Haake Minilab II (Thermo Scientific Haake GmbH, Karlsruhe, Germany), which consists of two co-rotating screws. This micro-compounder combines both melt processing operations and measurements of torque.

For each extrusion trial, 6 g of PLA and PBS granules, together with additives (dried if necessary), were manually mixed. Then they were dried in a vacuum oven at 60 °C for 6 h. Once the desired temperature was reached, the calibration of pressure transducers, before the beginning of measurements, was done. The mixture was fed into the co-rotating mini-extruder manually through a little hopper. After the introduction of the material, the melt, pushed by the screws, runs through a closed circuit (with the valve closed) for 1 min, during which the torque is measured as a function of time The material was recovered after one minute of rotation inside the chamber to ensure correct mixing. Acceptable values were obtained from at least ten experimental tests to guarantee the reliability and consistency of the test and to recover enough material for further tests. In the experiments, the rotating speed was 110 rpm, and the processing temperature was 190 °C. The final torque value represents the most significant value for the sample as the melt stabilizes. With the opening of the valve, the material was recovered in the form of a rectangular section wire and used for subsequent tests. The extrusion time, including the feeding operations, was, in total, 120 s to avoid degradation phenomena and all the data recorded by ThermoSoftware were elaborated to obtain the desired graphs.

After the preliminary study in miniextruder, films of about 100 microns of thickness and 20 mm of width were prepared by flat die extrusion. The films were prepared by mixing polymers, fillers and plasticizer into the twin screw extruder connected with a flat die. After drying PLA, PBS commercial pellets, calcium carbonate and PlastiStrength in a vacuum oven for 24 h, the films were produced through the COMAC EBC25HT semi-industrial co-rotating twin-screw extruder purchased from COMAC s.r.l, (Cerro Maggiore, Milan, Italy) connected to a AMUT flat die and calendering system located into the Pisa University laboratories of the Department of Civil and Industrial Engineering.

The extruder, equipped with two 25 mm co-rotating screws in a barrel with L/D = 44, has an integrated engine and a temperature control system that lowers the temperature using distilled water as the refrigerant and the pellets are loaded with a volumetric feeder. 

During the extrusion, several proofs were carried out to define the optimal temperature profile, considering temperatures between 150 and 180 °C, in order to avoid melt fracture instabilities. 

With the aim of maintaining the engine amperage such that neither the screws worked unloaded nor with an excessive flow of material, an empirical balance between the flow rate of the granules and the screws speed was carried out. When fully operational, the screws rate was maintained at 270 rpm, mass feed at 10 kg/h for polymers and fillers and 2 kg/h for plasticizer.

The material that comes out from the die in the form of a molten plate goes immediately in contact with a system of thermostatic rollers to allow cooling and solidification. Due to the motion of the rollers, the film undergoes elongation with a consequent reduction in thickness. 

#### 2.2.2. Characterization of Blends 

##### Melt Flow Rate

The investigation of flow behavior was carried out with CEAST Melt Flow Tester M20 (Instron, Canton, MA, USA) equipped with an encoder. In this case, the instrument measures the melt volume rate (MVR) of the polymer acquired by the encoder that follows the movement of the piston. Each polymer mixture obtained from the micro-compounder was shredded and dried in an oven at 60 °C for one day and used for tests, carried out in two replicate. The melt flow rate (MFR) is defined as the weight of molten polymer passed in 10 min through a capillary of specific diameter and length by pressure applied through a weight following the ISO 1133:2005. In the following work the standard used is the ISO1133D custom TTT: The sample is preheated without weight for 40 s at 190 °C, then a weight of 2,160 kg is released on the piston and after 5 s a blade cuts the spindle starting the real test. Through the encoder, every 3 s, an MVR measurement is recorded.

All the MVR data are reported indicating their standard deviations provided by the CEAST software. The MFR values standard deviations were calculated by considering results obtained by three measurements.

##### Mechanical Testing

From the materials prepared by Mini-extruder, films were prepared by compression molding starting from the flat wire recovered by the micro-compounder and pressing them at 180 °C for 30 s without applying any additional pressure and cooling with compressed air. In this way, the direction of preferential orientation given to the material by the extrusion was preserved to simulate in the tensile test the behavior of the film obtained in flat die extrusion. Tensile tests, performed on ISO 527-2/5A dumbbell specimens obtained with a Manual Cutting Press EP 08 (Elastocon, Brahmult, Sweden) were carried out in machine direction by an Instron universal testing machine model 5500R (Canton, MA, USA). The machine was equipped with a 100 N load cell, compressed air grip and interfaced with a MERLIN software (INSTRON version 4.42 S/N–014733H). The initial grip separation was 25 mm, and the deformation rate was set at 100 mm/min.

This kind of test was carried out on the dumbbell specimens, cut from semi-industrial films obtained from the flat die extrusion.

On those films also trouser tear tests, following the ASTM D1938, were carried out by a Universal Testing Machine INSTRON 5500r, Load Cell 100 N, Initial Grip Separation 50 mm, Grip Separation Rate 250 mm/min. Samples were cut in the machine direction (MD) and cross direction (CD) with a manual box cutter with a 45° blade from the top of the pre-crack to the bottom of trousers achieving legs dimensions 65 mm × 12.5 mm and an uncracked area of 25 mm × 25 mm. Before the test, the specimens were stored in a 50% humidity-controlled cell. At least ten specimens were tested for each sample, and the average values reported.

All the mechanical data are reported in this paper, indicating their standard deviations.

##### Scanning Electron Microscopy (SEM)

Morphological analysis on extruded filaments of PLA/PBS blends was done using FEI Quanta 450 FEG scanning electron microscope (SEM) (Thermo Fisher Scientific, Waltham, MA, USA). The cryo-fractured surface was covered with a tiny metallic layer of Au, in a way that the surface could be electrically conductive.

##### Differential Scanning Calorimetry (DSC)

The thermal analysis of samples was performed using DSC TA Instruments Q200 (TA Instruments, New Castle, UK) with RSC cooling system. The DSC analysis was run under nitrogen conditions, because air could oxidize the materials, and using two sealed containers on samples of about 10 mg.

The standby temperature of the cell is set at 40 °C and the flow of nitrogen in the cell at 50 mL/min. The method set to perform the thermal analysis was: Jump up to −40 °C; Ramp up to 220 °C, at 10 °C/min; Isotherm for 2 min at 220 °C; Decrease up to −40 °C, at 10 °C/min; Ramp up to 200 °C, at 10 °C/min; Jump up to 40 °C. By using the TA Universal Analysis software, it is possible to determine glass transition temperature (T_g_), melting temperature ©, cold crystallization temperature (T_cc_) and melting enthalpy (ΔH_m_).

In addition, the percentage of crystallinity of PLA X_c_ can be obtained (in our case of the PLA) through the following relation:

$$X_{C}=\frac{{\Delta H}_{m,\mathrm{PLA}}-{\Delta H}_{C,\mathrm{PLA}}}{X\cdot {\Delta H}_{\mathrm{PLA}}^{0}}\cdot 100,$$

where ${\Delta H}_{m,\mathrm{PLA}}\;$and ${\Delta H}_{C,\mathrm{PLA}}\;$are the melting enthalpy and the enthalpy of cold crystallization of PLA obtained in J/g, X is the weight fraction of the species that crystallizes (in our case PLA) and ${\Delta H}_{\mathrm{PLA}}^{0}\;$is the melting enthalpy of the 100% crystalline PLA, equal to 93 J/g [61].

##### Infrared Spectroscopy

Infrared spectra were recorded in the 550–4000 cm^−1^ range with a Nicolet 380 Thermo Corporation Fourier Transform Infrared (FTIR) Spectrometer (Thermo Fisher Scientific, Waltham, MA, USA) equipped with smart Itx ATR (Attenuated Total Reflection) accessory with a diamond plate, collecting 256 scans at 4 cm^−1^ resolution. ONMIC software was used to modify the intensity of spectra and for shifting them to obtain the normalization with respect to selected reference bands, for a better comparison of spectra profiles. The spectra were recorded onto films prepared by PLA and PS acrylic copolymer dissolved in chloroform.

##### In Vitro Biocompatibility Tests

Cytocompatibility tests were carried out by using hMSCs and human immortalized dermal keratinocyte (HaCaT cell lines). It was assumed that hMSCs are a representative cellular model for connective tissues, such as dermis, and that keratinocytes can predict behavior of epidermis, according to antibacterial and anti-inflammatory response [62]. The different films, including polyethylene (LDPE) film as a commercial control, were sterilized overnight in ethanol, rinsed three times with DPBS, and placed on the bottom of 6-well plates. HMSCs were cultured using DMEM low glucose supplemented with 2 mM L-glutamine, 100 IU/mL penicillin, 100 mg/mL streptomycin and 10% heat-inactivated FBS for eight days on the films. HaCaT cells were cultured in DMEM supplemented with 1% Pen-Strep, 1% glutamine and 10% fetal calf serum in 12-well plates until 80% of confluence, were incubated for 24 h with the films. All cell culture studies were performed in a humidified incubator at 37 °C in air and 5% CO_2_.

AlamarBlue^®^ test was performed under UV-VIS spectrophotometry at days 1, 4 and 8 to monitor hMSC viability in 24 well plates (*n* = 6) following the manufacturer’s instructions [56].

HaCaT cells were incubated with the films for 6 h and 24 h. At the end of the experiment, the mRNA was extracted from the cells and the levels of expression of the proinflammatory cytokines, interleukins (IL)IL-8, IL-6, IL-1α IL-1β and tumor necrosis factor alfa (TNF-α), the anti-inflammatory cytokine transforming growth factor beta (TGF-β) and the antimicrobial peptide human beta defensin 2 (HBD-2) were evaluated by Real-Time polymer chain reaction (PCR).

To evaluate the anti-inflammatory and indirect antibacterial activity of the films, HaCaT cells were seeded in 6-well culture dishes until 80% of confluence and placed with F3, LDPE and PLA for 6 and 24 h.

At the end of the experiment, total RNA was isolated and reverse-transcribed into cDNA (1 mg per sample) using random hexamer primers at 37 °C for 60 min, according to the manufacturer’s instructions. Real time PCR was performed using LC Fast Start DNA Master SYBR Green kit, using 10 ng of total RNA in a 20 µL final volume, 3 mM MgCl_2_ and 0.5 µM of sense primer and antisense primers (Table 2).

## 3. Results

### 3.1. Plasticized PLA/PBS Blends Processability and Properties

#### 3.1.1. Investigation about PLA/PBS Extrusion by Micro-Compounding and Melt Fluidity Tests

Regarding pure PLA, it was observed that, although it was preliminarily dried, it underwent some chain scission during extrusion, as shown by MFR and MVR data in Table 3. The addition of 2% of PS acrylic copolymer-based product only slightly affected the MFR and MVR values, but resulted in a significant increase in torque. Plasticized PLA/PBS blends were prepared by miniextrusion with the addition of PS and micro calcium carbonate 2AV (Table 1). The torque increased with the addition of PS, and at the same time, the MFR and MVR decreased by adding the melt strength enhancer and calcium carbonate. The increase in viscosity, due to the addition of the calcium carbonate, is more evident considering the MFR/MVR values, and can be attributed to the occurrence of interactions between the matrix and the filler, which represent a sort of physical cross-linker of the mixture. The reference blend F1 has a low torque value and a high MFR with respect to pure PLA due above all to the high percentage of liquid plasticizer, which makes the mixture very fluid in the melt. The addition of PS in F2 is necessary to increase the torque and decrease the MFR. With the addition of calcium carbonate at 4% (F3) the torque increased with respect to the F1 formulation but not appreciably with respect to the F2 blend.

**Figure 1:**
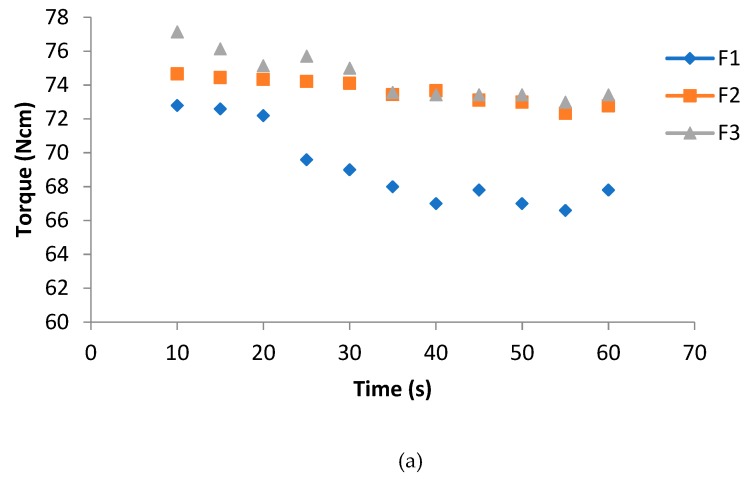

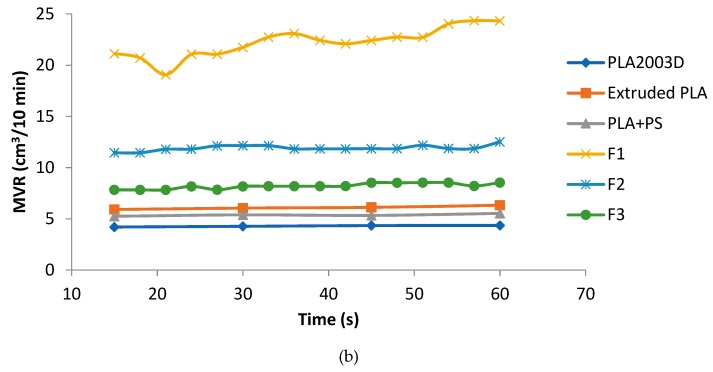
(**a**) Torque as a function of time; (**b**) MVR as a function of time.

The trend of the torque decreased as a function of time, due to the addition of plasticizer in the blends; on the other hand, PS enhanced the melt viscosity stability (Figure 1a). The trend of the MVR better showed the difference between F2 and F3. It can be seen how the plasticizer alone makes the trend unstable. The simultaneous addition of PS and calcium carbonate leads the material to have a trend similar to the pure extruded PLA (Figure 1b).

The improvement of PLA melt strength achieved thanks to acrylic copolymer-based products (like those of PS) is not fully explained, but significant increase in melt viscosity through rheology tests were evidenced and attributed to the combination of high miscibility and interactions between PLA and the copolymers [55]. To better understand the nature of the interactions between PLA and PS acrylic copolymer, some specific investigations were carried out by infrared and DSC measurements. For this specific study, different films were prepared by solvent casting, to maximize intermolecular interactions, adding from 5% to 20% of PS to PLA (Table 4). PLA and PS acrylic copolymer-based product were completely dissolved in the minimum amount of chloroform, and then the mixture was poured in a Petri dish. All the prepared samples were left under a fume hood for at least 48 h at room temperature to eliminate the solvent.

**Figure 2:**
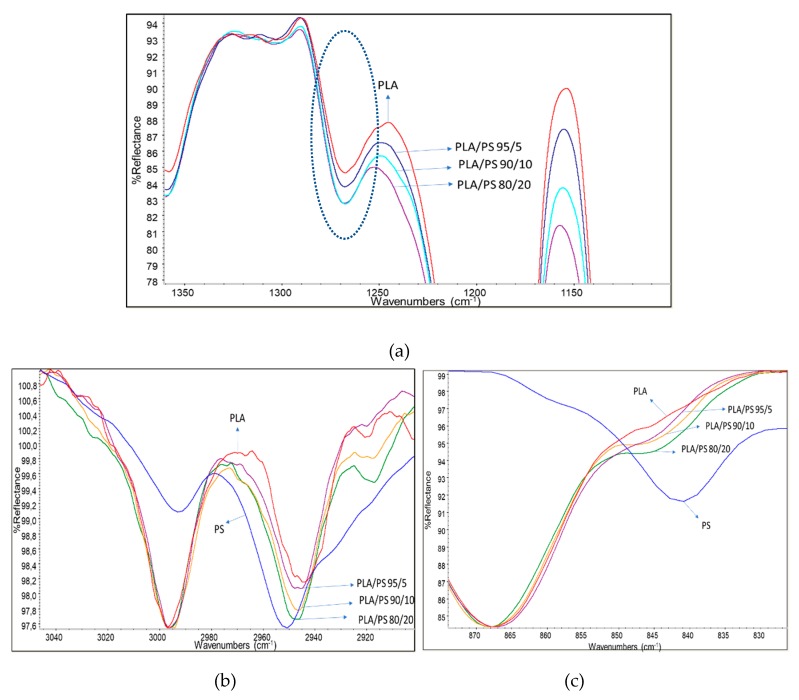
(**a**) zoom onto the amorphous band of PLA; (**b**) C-H stretching region of the spectra; (**c**) rocking CH_2_ band (typical of poly(methyl methacrylate)—PMMA) region of the spectra. Spectra were slightly shifted on the vertical direction for normalizing with respect to reference bands, so the reflectance intensity is expressed in arbitrary units.

Films were characterized by infrared spectroscopy in ATR mode (Figure 2). According to Meaurio et al. [63], in the region characteristic of the stretching of the C–O group between 1200 and 1300 cm^−1^, it is possible to observe the band attributable to the amorphous fraction of the PLA (Figure 2a). The pure PLA spectrum showed the less intense band in this range. By increasing the amount of PS acrylic copolymer-based product in the blend the band intensity increased, revealing the increase of the amorphous fraction in PLA. The presence of PS seems, thus, to obstacle the PLA crystallization, because of its homogenous distribution in the film. Thus, despite its low content (2%), the presence of the acrylic chains has a significant effect on chains mobility.

**Figure 3:**
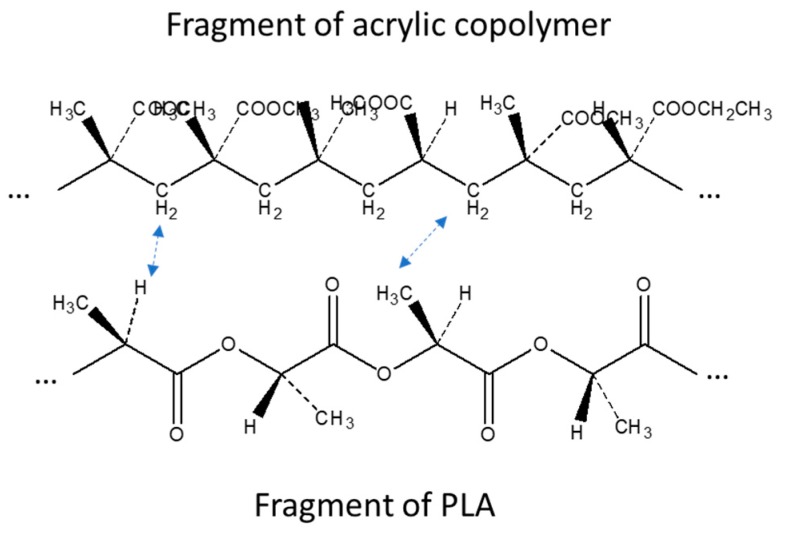
Interactions between PLA and PS acrylic copolymer as deducted by ATR-IR study.

Other interesting changes with respect to the PLA spectrum, not simply explainable by the superimposition of PS acrylic copolymer bands, were found in the CH stretching region (Figure 2b). In fact, although in this part of the spectrum signals are weak, the addition of PS slightly shifted the peak relative to the asymmetric stretching of CH_2_ from 2945 cm^−1^ of pure PLA to 2950 cm^−1^ of the melt enhancer, indicating some possible interactions of the CH_2_ of PS with the CH_3_ of PLA. Moreover, the weak peak at 2920 cm^−1^, attributable to the stretching of the C–H group of PLA, was slightly shifted to lower wavenumbers, due to the presence of increasing amounts of PS. In Figure 2c, on the other hand, it is possible to observe how the rocking CH_2_-related shoulder of PS [64] moves towards lower wavelengths by increasing the percentage of PS in the mixture. As the carbonyl bands at 1745 cm^−1^ did not show peculiar shift, due to the addition of PS, and also the typical bands of C-O did not show appreciable changes, this study confirms that PLA interacts with PS acrylic copolymer because of the peculiar structure of this copolymer, having polar groups on one side and non-polar CH on the opposite side with respect to the polymer chain (Figure 3). This result is quite surprising, because electronegative oxygen atoms are present in macromolecules, and polar bonds are stronger than van der Waals bonds; hence, the dipole interactions between macromolecules should be not negligible. We can tentatively explain this result considering that these kinds of dipole-dipole interactions between a PLA and a PS macromolecule are very similar to the inter-macromolecular interactions occurring between two PLA macromolecules or two PS macromolecules, whereas, the interactions between CH, CH_2_ and CH_3_ groups can be much affected by the specific orientation of groups with respect to the main carbon chain in PS acrylic copolymer and PLA chains (Figure 3).

The films were also maintained at 190 °C in the compression molding press for 2 min to simulate the processing of the blends, with the aim of controlling if the thermal treatment can induce chemical modifications in the polymers. The infrared spectra recorded on these treated samples showed a perfect overlap with samples not treated in the press that suggest the non-formation of new bonds between PLA and PS acrylic copolymer product.

**Figure 4:**
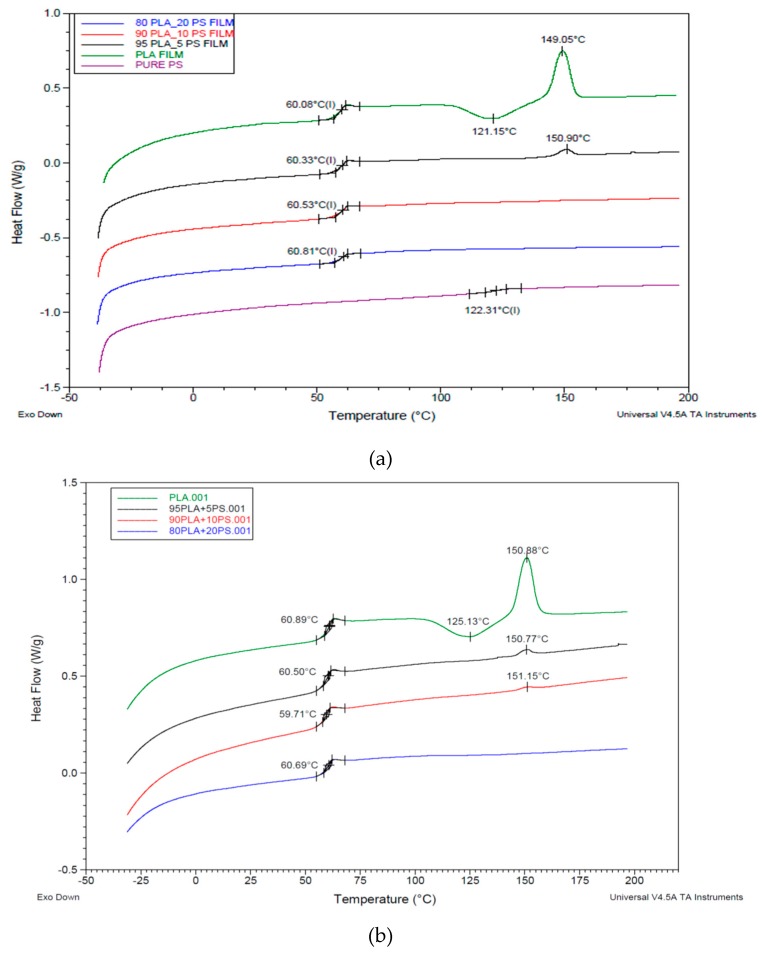
Comparison between the second heating thermograms of the (**a**) PLA/acrylic copolymer-based product (PS) cast films; (**b**) PLA/PS cast films treated for 2 min at 190 °C. Exothermal peaks are directed down.

The PLA/PS films were also analyzed by DSC. The pure PS showed a glass transition temperature at 122 °C, but in the blend, its glass transition completely disappeared, and only one glass transition at 60 °C could be observed (Figure 4a). An amount of 5% of PS was enough to inhibit the crystallization of PLA but did not affect the glass transition of the material. It is also possible to observe how 20% of PS did not influence the glass transition temperature of the blend, despite this polyacrylate has a T_g_ of about 120 °C (very similar to the one of poly(methyl methacrylate) (PMMA)). However, according to Samuel et al. [65], the amount of PMMA needed to influence the glass transition temperature of the PLA/PMMA miscible mixture is higher than 20%. The DSC data confirmed, in general, the good miscibility and the presence of not negligible interactions between PS with PLA. Hence, although the amount of PS added in the melt during the extrusion of PLA is relatively low, its good miscibility can mainly explain its effectiveness as melt strength regulator, despite the absence of reactive groups. The decrease of melt viscosity, due to PLA chain scission, is thus counterbalanced by the occurring of interactions between PLA and PS acrylic copolymer-based product homogenously distributed in the melt.

After the thermal treatment at 190 °C in the compression molding press no evidence of structural changes, e.g., due to the formation of new bonds, could be evidenced (Figure 4b). In fact, the glass transition temperatures, as well as the crystallization and melting temperature, did not change significantly comparing the thermograms.

#### 3.1.2. Tensile Properties

Tensile properties were measured onto compression molded films of F1, F2 and F3 microextruded blends. The addition of PS acrylic copolymer-based product in F2, improved the stress at the break but at the same time decreased the elongation at break. Compared to F2, the addition of calcium carbonate at 4% did not induce a decrease in mechanical properties. The carbonate particles could form aggregates where the rupture could propagate, favoring the breakage of the specimens. Moreover, the presence of the filler interacting with polymeric chains make their sliding more difficult, and the elongation values result lowered. The addition of carbonate induced elongation and stress values comparable to the F1 and F2 blends. Therefore, the tensile properties of plasticized PLA/PBS blends remain good even after the addition of PS acrylic copolymer-based product and 2AV in the explored composition range. It can be observed that the reinforcement introduced in these blends led to the modification of the first part of the stress/strain curve respect F1. The interactions between filler and matrix made the materials less deformable in the initial part of the curve. The addition of PS and 2AV led to the increase in the yield stress compared to the F1 mixture, which exhibited a behavior quite similar to an elastomer (Figure 4). However, in general, the addition of PS and 2AV only slightly modified the plasticized blend behavior, typical of a flexible film with high elongation at break.

#### 3.1.3. DSC Characterization

**Figure 5:**
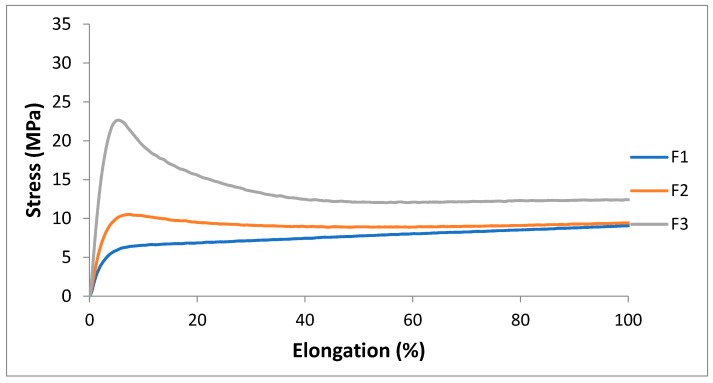
The first part of stress/strain curves (up to 100% of elongation) of F1, F2, F3.

The analysis of the first heating scan in the DSC thermograms of the films revealed the thermal history of the films, rapidly cooled after compression molding. The melting temperatures of all the samples are comparable (the maximum difference is 2 °C) (Table 5). Despite the rapid cooling, a significant crystalline fraction was present in the blends. A decreasing trend of the film crystallinity passing from the reference plasticized blend F1 to the blends in which the additives are present was observed. This showed how the addition of PS acrylic copolymer-based product discouraged the formation of crystals during the rapid cooling after compression molding, as also confirmed by Kaczmarek et al. [66]. For F1 the T_g_ is 44°C, and with the addition of PS and 2AV the T_g_ decreased up to 30 °C. This behavior can be attributed to the peculiar profile of the thermogram (Figure 5) with an enthalpic relaxation peak above the glass transition attributed to aging [38] that was not present in F2 and F3, where the aging was, thus, prevented thanks to the presence of the additives. Interestingly, in the presence of the filler and the melt strength enhancer, the exothermic crystallization peaks shifted at lower temperature, and the value of the ΔH_c_ of F1 was lower than F2 and F3. Thus, the presence of the two additives facilitated the cold crystallization during heating of PLA.

**Figure 6:**
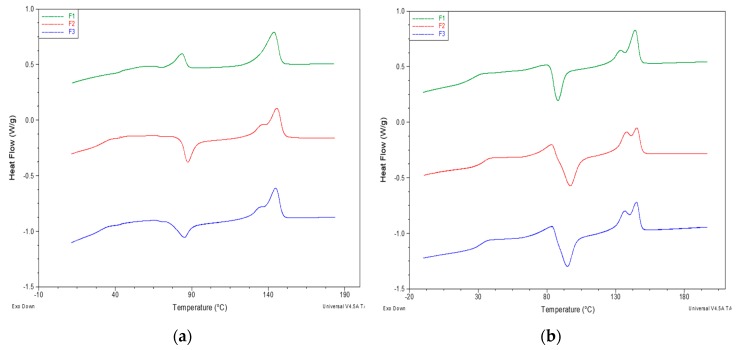
DSC heating thermograms of F1, F2 and F3: (**a**) First heating; (**b**) second heating. Exothermal peaks are oriented down.

In general, the trend of the thermogram was similar for F2 and F3 (Figure 6a): With the increase in temperature above the glass transition temperature there is the formation of crystals (exothermic peak) and subsequently their melting. No evidence of the melting of PBS could be revealed in first heating thermograms.

Table 6 showed the values of glass transition temperature (T_g_), crystallization temperature (T_C_), crystallization enthalpy (ΔH_C_), melting temperature I, melting enthalpy (ΔH_m_) and crystallinity percentage (X_C_%) of the samples in the second heating, occurred after the controlled cooling step at 10 °C/min from the melt. The F1 blend showed a T_g_ of 27 °C, while F2 and F3 showed a glass transition at about 30 °C. The melting temperature was at about 144 °C for the three blends. Melting peaks of PBS were visible when PS acrylic copolymer and 2AV were added, indicating a potential nucleating effect of the additives. Similarly, the PLA cold crystallization peak could be observed only for these two samples.

In the second heating thermograms (Table 7), thanks to the plasticization of the PLA, its glass transition temperature moves towards lower values, compared to 60 °C of the pure polymer. Moreover, the addition of PS and 2AV led to an increase in the PBS melting enthalpy. The addition of the melt strength enhancer (F2) provided the highest value of cold crystallization enthalpy and the lowest of crystallinity. Probably the acrylic compound promoted intermolecular interactions between the polymer chains that prevent the formation of crystals during cooling, while the addition of a filler increases the number of crystalline germs from which the crystal can grow.

Interestingly, the cold crystallization occurred for F2 and F3 blends more extensively and in a larger range of temperatures than in F1. Correspondingly, the melting peak at a lower temperature, typical of less regular α’ crystals [7], increased (Figure 6b).

#### 3.1.4. Phase Morphology Study

**Figure 7:**
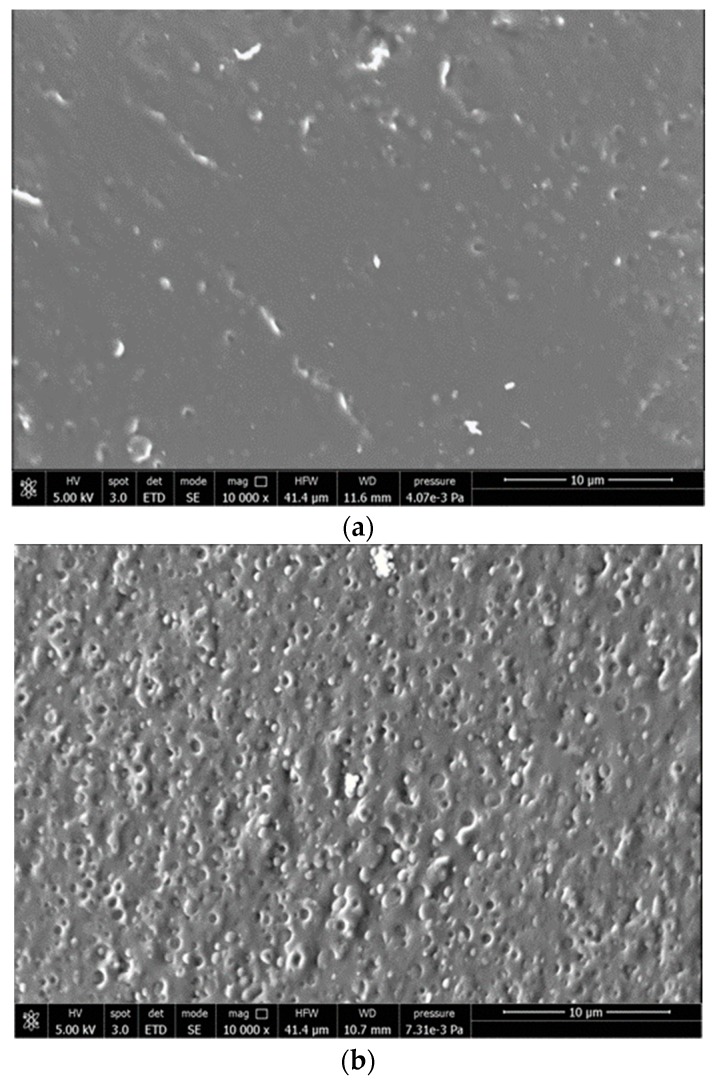

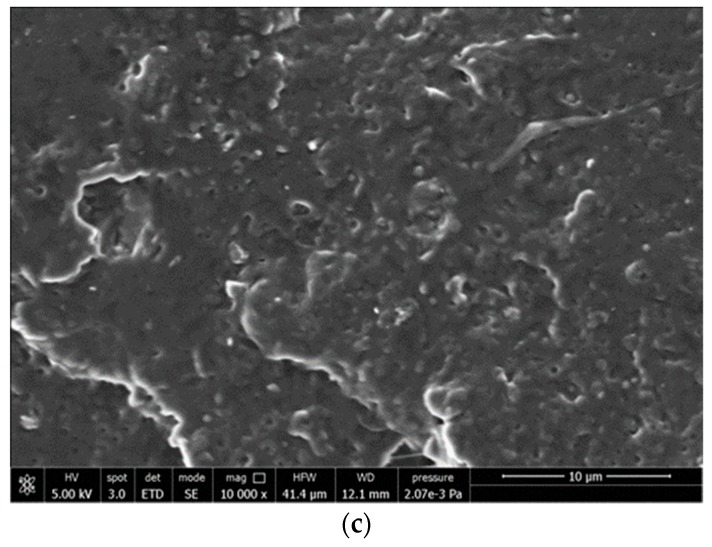
SEM micrographs recorded on cryo-fractured surfaces of (**a**) F1; (**b**) F2; (**c**) F3.

The main components of the F1, F2 and F3 compounds are PLA and PBS in an 80/20 ratio and ATBC, that is highly miscible in PLA blends [29] (Table 1). The three PLA/PBS blends, analyzed by SEM, showed a morphology typical of immiscible polymers with high compatibility (Figure 7). In fact, they were characterized by PBS almost spherical domains of sub-micrometric dimension dispersed quite uniformly as droplets into the PLA matrix. The PBS particles appear well defined on the cryogenic fracture surface, and no significant differences could be evidenced, due to the addition of PS acrylic copolymer-based product and 2AV. Hence, these additives, that represent only a minor fraction in the material, and could not be revealed, did not significantly modify the phase morphology typical of the plasticized PLA/PBS blend.

### 3.2. Plasticized PLA/PBS Films by Flat Die Extrusion

#### 3.2.1. Mechanical Properties of Flat Die Extruded Film

Regarding the mechanical properties of the flat die extruded films, the tests were carried out on ISO 527-2-5a dumbbell specimens with a thickness of 100 µm in the machine and in cross direction, that means along the flow direction collected by the wind-up roll and perpendicularly.

**Figure 8:**
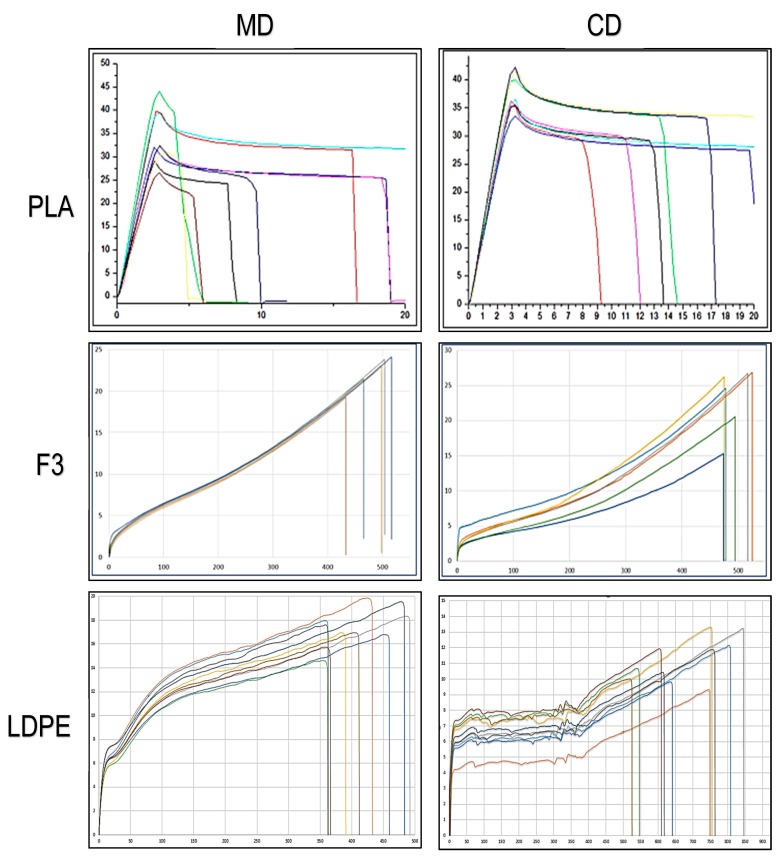
Stress/strain curves for PLAF3 and LDPE dumbbell taken from flat die films. On the vertical axis the stress in MPa is reported and on the horizontal axis the percentage of elongation.

The results (Table 8) and the curves (Figure 8) obtained for F3 formulations were compared with flat die extruded films of pure PLA to evidence the improvement in flexibility. Moreover, they were also compared with flat die extruded films of LDPE with the same temperature profile (to guarantee a correct processing comparison) taken as a reference of current fossil-based alternatives. In fact, the curves in the MD and CD for F3 have the typical appearance of ductile films that undergo uniaxial elongation, “rubber-like” [67]. At low stresses, they exhibited a linear viscoelastic response although it would be more correct to state that the behavior of stress-strain curves is rubber-like. Therefore, the response is non-linear even at low stresses [68] where there is no constant value of the elastic modulus, i.e., σ = E∙ε is only true if E is a variable. In the literature, some writers also disagree with the definition of yield when there is the switching to strain-hardening behavior at higher deformation for films mechanical properties analysis [69].

In this paper, however, the elastic module has been intended as the linear proportionality that occurs at low stress values after the specimen has settled at the beginning of the test and the yielding stress is referred to the knee of the curve, and breaking stress correspond to the maximum stress.

The two graphs of the MD and CD for F3, looking at the shape, are very similar each other (with less scatter for the cross direction) also as far as concern mechanical values and comparable with typical quasi-static mechanical properties of commercial biodegradable films [70,71].

In agreement with the results of tensile tests performed onto films produced by miniextrusion, this new material can be considered “rubber-like” thanks to the quantity of plasticizer and PBS added to the PLA matrix. It showed an elastic modulus of about 0.1 GPa and high values of stress at break and, above all, elongation at break, a fundamental property to evaluate the films resistance, slightly greater in machine direction where it reaches values of about 500%.

The flexibility value results, inversely correlated to the value of E, in the machine direction, resulted even greater than LDPE tested as a reference, and also resulted with higher mechanical resistance (σ_b_). LDPE dumbbell specimens cut from flat die films show, instead, a very good ductility in cross direction, as the elongation at break is the highest recorded in this work.

**Figure 9:**
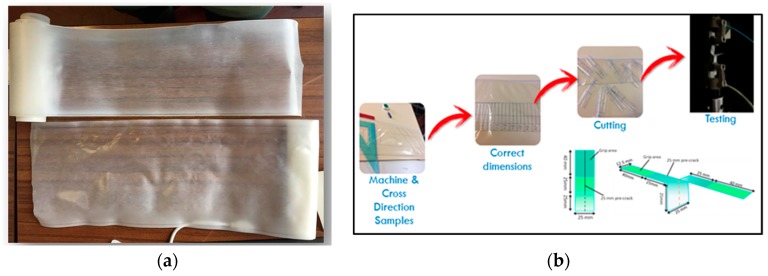
(**a**) F3 semitransparent films obtained by flat die extrusion; (**b**) methodology adopted for trouser tear tests.

Not only flexibility needs to be improved to ensure a good film formulation, but also its toughness. In most cases, these films break out of mode III out-of-plane and a technique to evaluate resistance to this type of fracture is the trouser tear test [72,73]. It is a common technique to measure the critical fracture energy during fracture of films; the origin of the name is correlated to the trouser shape of the samples. The legs of the trouser are pulled in the opposite direction to create the tearing action (Figure 9)

In Equation (1), adopted in this work, Critical Fracture Energy, T, (N/m) is calculated for the case where the legs are not stretched during the test. (F is the load, λ t is the thickness,

(1) $$T=\frac{2F}{t}$$

In Table 9, it can be noticed that in the MD, the values of critical fracture energy absorbed by F3 is two times higher than pure PLA in the MD and three more times in cross direction. The orientation of the flow opposite to the direction of crack propagation allows the crestion of greater obstacles to the propagation of the crack itself, making the film more tough and capable to absorb more energy in the CD before the breakage.

The value obtained for F3 is significantly lower than the values observed for LDPE, the current fossil-based alternative for films used, for example, in films in contact with skin. In any case, the achieved improvement, much evident in tensile properties (Table 8 and Figure 8), can be enough for films suitable for several applications.

#### 3.2.2. Compatibility with Cells of LDPE, PLA and Plasticized PLA/PBS Films

**Figure 10:**
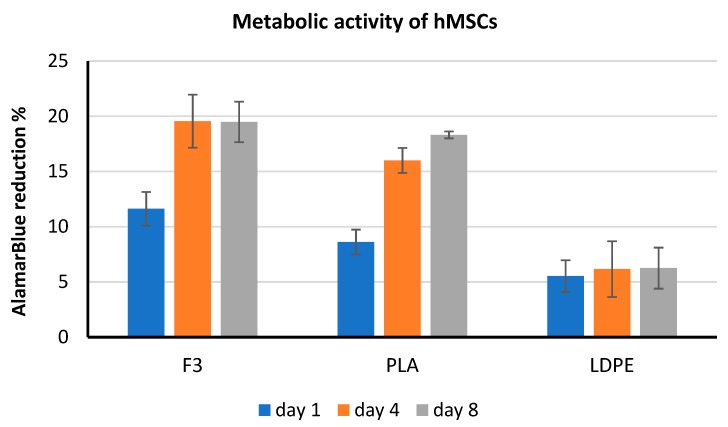
Results of AlamarBlue^®^ test performed with hMSCs.

In vitro compatibility studies were performed using hMSCs via the AlamarBlue^®^ metabolic activity assay. This test showed that films made with formulation F3 and pure PLA resulted in more cytocompatible than the LDPE, by showing a four-time increase of cell viability on day 8 (Figure 10). AlamarBlue^®^ assay was qualitatively performed also on HaCaT cells after one day of exposure to the films, corroborating the results obtained with hMSCs, in which F3 was the most performing substrate.

**Figure 11:**
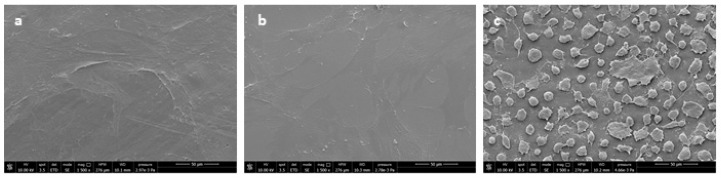
SEM micrographs of films cultured in contact with hMSCs: (**a**) F3, (**b**) PLA, (**c**) LDPE.

SEM analysis of hMSCs cultured on the samples (Figure 11) showed that on LDPE films, cells maintained a round morphology, a sign of difficult adhesion, whereas, on F3 and PLA films, cells spread and completely attached to the surface.

**Figure 12:**
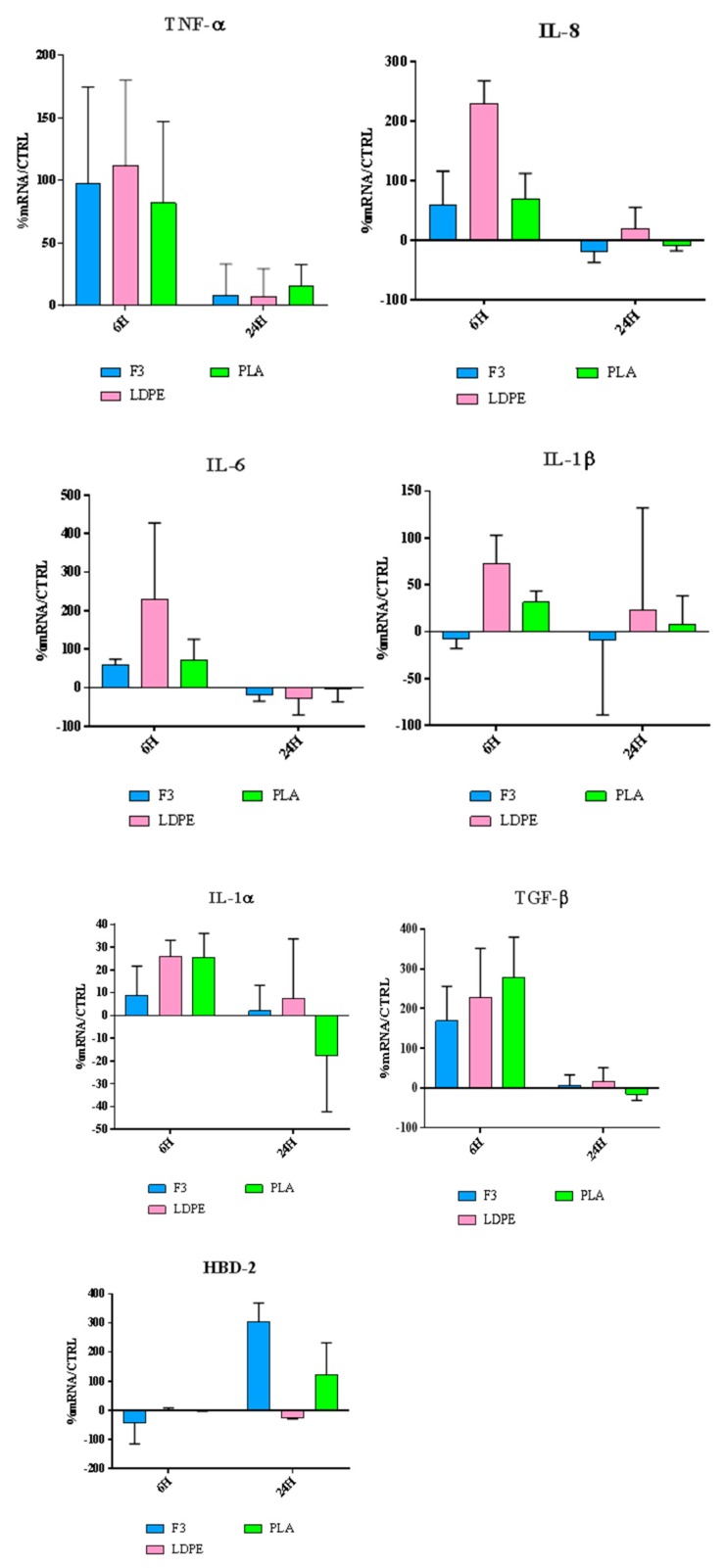
Relative gene expression of proinflammatory cytokines and HBD-2 in HaCaT cells. Data are expressed as mean ± SD as a percentage of the relative mRNAs in each group compared to plain HaCaT cells as a control.

For contact with skin, short term response by keratinocytes is very important, as it can predict the pro-inflammatory behaviour of materials and ultimately, the possibility of an innate immune response by the production of antimicrobial peptides, like defensins. HaCaT cells were exposed to the produced films, and the expression of different cytokines was evaluated at the gene expression level (Figure 12).

Our results (Figure 12) show that all the films F3, LDPE and PLA possess a significant immunomodulatory activity. In fact, they are capable of upregulating the expression of both pro-and anti-inflammatory cytokines. This behavior can be explained by giving the films a role in the wound repair process, as better explained in the discussion part.

## 4. Discussion

The use of biobased plasticizers is a valuable strategy to decrease PLA-based blends rigidity and increase its renewable content. As a proof of concept, the blend F1 showed very good flexibility and a high value of elongation at break. On the other hand, the addition of the liquid plasticizer to the blend provoked a strong decrease in melt viscosity. Therefore, this material was not suitable for flat die extrusion. In fact, after the exit from the flat die, the material in the form of a viscous liquid, must have the necessary melt strength to be stretched by the calendaring system. If the viscosity is too low, this operation is not possible. The inter-macromolecular interactions promoted by the acrylic copolymers of PS product in the PLA-based blends were, thus, fundamental for achieving this result. The PS acrylic copolymer-based product interacted with the PLA chains thanks to its good miscibility and increased resistance in the melt and at the same time improved the mechanical resistance. The addition of this copolymer inhibited the crystallization of PLA without affecting the glass transition temperature of the material.

The thermal properties were modified by the presence of PS acrylic copolymer-based product, in agreement with the experiments made onto casted films consisting of only PLA and PS. The presence of PS acrylic copolymer homogeneously dispersed in the PLA-based blend hindered the formation of crystals both during the rapid cooling and during the controlled cooling. Interestingly, by also adding 2AV (F3), behaving as a slight nucleating agent, the final value of crystallinity resulted almost independent on the cooling procedure, as deductible by comparing the first heating with the second heating data.

The use of the plasticizer alone gave rise to low viscosity and low melt resistance of the blends, but it increased the flexibility of the samples. The mechanical properties, typical of a flexible rubbery-like system, were only slightly affected by the addition of PS acrylic copolymer and 2AV. However, the improvement of inter-macromolecular interactions as evidenced by the slight but not negligible increase in stress at yield.

F1-based films could not be produced by flat die extrusion, whereas, F2 resulted in sticky on the surface. The flat die extrusion of F3 was suitable in terms of processability, and the extruded films resulted highly flexible with respect to pure PLA with high elongation at break. The tear resistance was significantly enhanced with respect to that of PLA both in the MD and CD. However, the tear resistance remained lower than that of LDPE.

The in vitro biocompatibility tests demonstrated that PLA and F3 films favored the adhesion and growth of hMSCs, much better than LDPE. Moreover, similar results were obtained with keratinocytes, representative of epithelial cells of the skin.

Our results show that all the films F3, LDPE and PLA possess a significant immunomodulatory activity, in fact, they are capable of upregulating the expression of both pro-and anti-inflammatory cytokines. This behavior can be explained by giving the films a role in the wound repair process.

In fact, wound healing is characterized by two main phases: In the first phase a number of overlapping events occurs, including the production of pro-inflammatory cytokines. IL-1α and TNF-α represent the primary cytokines for pro-inflammatory responses. A direct effect of IL-1 release is the upregulation of IL-6 and IL-8 production, chemokine with angiogenic properties.

The second phase is associated with growth-oriented cytokines and factors, among which TGF-β. This anti-inflammatory cytokine has multiple functions throughout wound healing, including playing a critical role in the deposition of extracellular matrix proteins and preventing hyperproliferation of keratinocytes after wound closure.

Furthermore, F3 and PLA can stimulate, after 24 h of incubation, HBD-2 production; therefore, we can hypothesize that they are potentially endowed with an indirect antimicrobial activity [74,75,76].

It is hypothesized that PLA-based materials stimulate the self-defense of cells against microbial agents and simultaneously increase cellular viability by virtue of their biocompatible nature. A similar but more effective action, was shown by some natural anti-microbial bio-polymeric agent, like chitosan/chitin [34,35,62]. The slight antimicrobic action of PLA-based blends can be related to the slight formation of lactic acid. In fact, at the temperature of the body, implanted PLA is naturally degraded over time into well-tolerated and safe degradation products, which are secreted from the body [77].

In aqueous solutions, the hydrolytic degradation of PLA proceeds via random cleavage of the ester bond [78,79]. In general, the hydrolytic degradation of PLA-based solid polymer matrices can proceed through under two different mechanisms: Surface or heterogeneous reactions and bulk or homogeneous erosion [80]. In the case of textured films, the former mechanism is certainly favored.

Mutsuga et al. [81] had effectively demonstrated the migration of lactic acid, lactide and oligomers from PLA films in water under various temperature conditions. The tested conditions, in terms of temperature and pH, can be considered similar to those typical of the skin in contact with a plastic film. The generated monomers, which are carboxylic acids (lactic acids and its oligomers) accelerate polymer degradation by further lowering the pH [82]. Lactic acid and its oligomers can show a mild anti-microbial action. In fact, it is reported that Lactic acid showed antimicrobial activity against the primary *bacterial vaginosis* (BV) pathogen Gardnerella vaginalis, with a MIC of 3.6 mg/mL. In addition, biocompatible hydrogels are used for the vaginal administration of lactic acid to prevent and treat BV [83]. These considerations make the use of PLA-based formulation appealing for personal care and sanitary products in contact with skin.

In general, there are multiple factors that may influence cell interaction with biomaterial surfaces, such as morphological (e.g., micro/nanostructure, porosity, roughness), physical (e.g., hydrophilicity/hydrophobicity, mechanical properties) and also chemical (e.g., chemical groups and biomolecules present on the surface); therefore, it is difficult to identify one responsible factor for our biological outcomes. In fact, in our system, the composition of the materials was modulated to achieve the desired properties, and the addition of several ingredients was necessary for proficient film processing. Moreover, CaCO_3_ is present in F3 with a reduced weight ratio (4%) with respect to other additives, which may also concur to the obtainment of the final cellular response. It was, thus, investigated if CaCO_3_ could influence the cell/material interface through a change of the local pH. Such films are indeed conceived to be placed in contact with the skin, therefore, in a potentially humid microenvironment. We could observe that the pH generated by immersion of LDPE, PLA and F3 films in a small amount of deionized water (1 cm^2^/mL) were 6.5 ± 0.1, 4.9 ± 0.2 and 6.7± 0.2, respectively, indicating that CaCO_3_ in F3 composition exerted a modulatory effect on local acidity generated by plain PLA. However, the pH in the culture media used in cell culture experiments is maintained by an internal buffer system. Therefore, local pH variation, due to CaCO_3_, could not influence the biological response reported in this study. In good agreement, specific tests, based on pH measurements, made with the same methodology but in a McCoy’s reference culture media [84] resulted in a value of 7.99 ± 0.09, 8.07 ± 0.01 and 8.04 ± 0.01 for LDPE, PLA and F3, respectively. For this reason, it is reasonable that other factors, such as material mechanical properties and chemical composition (including, but not limited to CaCO_3_) of F3, altogether, could have played a role in the obtained biological results.

## 5. Conclusions

PLA/PBS-based blends were investigated both on a laboratory scale and on a semi-industrial scale. By considering the effect of different additives, selected blends were processed by using flat die extrusion to obtain biocompatible films. Processability and thermo-mechanical properties were evaluated, since they are greatly important for industrial fabrication. The improvement of PLA properties was possible using additives, such as a plasticizer, a melt strengthening agent (i.e., PS) and an inorganic filler (i.e., calcium carbonate). The introduction of the PS acrylic copolymer-based product and calcium carbonate in these blends led to the increase in melt viscosity and in the yield stress because of the interactions between the additives and the PLA matrix. In the first heating of the DSC analysis, the addition of PS and calcium carbonate determined a decrease of the crystallinity. The observed effects are attributed to the interaction of the PS acrylic copolymer-based product with the PLA chains, preventing the formation of crystals, in good agreement with the results of the specific investigations carried out in this work on PLA/PS blends obtained by solution casting, to evidence the occurrence of such interactions by spectroscopic and thermal analysis.

The films produced by flat die extrusion showed improved flexibility, elongation at break at tear resistance with respect to pure PLA and resulted competitive in many performances with fossil-based polyolefins. Moreover, the PLA film and the blends showed higher biocompatibility tested by using keratinocytes and mesenchymal stromal cells, with respect to LDPE and a slight anti-microbial effect. These results can suggest the use of renewable and biodegradable PLA-based films in applications in contact with skin and the human body, especially in applications where a better impact on health and the environment is desirable.

## Figures and Tables

**Table 1:** Plasticized poly(lactic acid) (PLA)/poly(butylene succinate) (PBS) blends prepared by micro-compounder.

Blends	PLA (%wt)	PBS (%wt)	ATBC (%wt)	PS (%wt)	CaCO_3_ (%wt)
F1	63	17	20	0	0
F2	62	16	20	2	0
F3	59	15	20	2	4

**Table 2:** Gene, primers sequences and conditions regarding the expression of the proinflammatory cytokines.

Gene	Primers sequence	Conditions	Product Size (bp)
**IL-1 α**	5′-CATGTCAAATTTCACTGCTTCATCC-3′ 5′-GTCTCTGAATCAGAAATCCTTCTATC-3′	5″ at 95 °C, 8″ at 55 °C, 17″ at 72 °C for 45 cycles	421
**TNF-α**	5′-CAGAGGGAAGAGTTCCCCAG-3′ 5′-CCTTGGTCTGGTAGGAGACG-3′	5″at 95 °C, 6″ at 57 °C, 13″at 72 °C for 40 cycles	324
**HBD-2**	5′-GGATCCATGGGTATAGGCGATCCTGTTA-3′ 5′-AAGCTTCTCTGATGAGGGAGCCCTTTCT-3′	5″ at 94 °C, 6″ at 60 °C, 10″ at 72 °C for 50 cycles	198
**IL-6**	5′-ATGAACTCCTTCTCCACAAGCGC-3′ 5′-GAAGAGCCCTCAGGCTGGACTG-3′	5″ at 95 °C, 13″ at 56 °C, 25″ at 72 °C for 40 cycles	628
**IL-8**	5-ATGACTTCCAAGCTGGCCGTG -3′ 5-TGAATTCTCAGCCCTCTTCAAAAACTTCTC	5″ at 94 °C, 6″ at 55 °C, 12″ at 72 °C for 40 cycles	297
**TGF-β**	5′-CCGACTACTACGCCAAGGAGGTCAC-3′ 5′-AGGCCGGTTCATGCCATGAATGGTG-3′	5″at 94 °C, 9″ at 60 °C, 18″at 72 °C for 40 cycles	439
**IL-1 β**	5′-GCATCCAGCTACGAATCTCC-3′ 5′-CCACATTCAGCACAGGACTC-3′	5″at 95 °C, 14″ at 58 °C, 28″at 72 °C for 40 cycles	708

**Table 3:** Plasticized PLA/PBS samples torque, melt volume rate (MVR) and MFR.

Blends	Torque (N·cm)	MVR (cm^3^/10 min)	MFR (g/10 min)
PLA ^a^	-	4.4 ± 0,1	5.0 ± 1
PBS ^a^	-	10.3 ± 0.3	10.8 ± 0.3
Extruded PLA	161 ± 5	6.4 ± 0.5	6.7 ± 0.5
PLA + 2% PS	183 ± 4	5.6 ± 0,2	6.2 ± 0.4
F1	68 ± 5	22 ± 2	24 ± 2
F2	73 ± 6	11.8 ± 0.9	12.4 ± 0.9
F3	73 ± 9	8.7 ± 0.6	9.4 ± 0.6

^a^ PLA and PBS are the not processed granules of pure polymers.

**Table 4:** Formulation of PLA/PS acrylic copolymer-based product cast films for the study of interactions.

Sample	PLA %wt	PS %wt
1	100	-
2	95	5
3	90	10
4	80	20

**Table 5:** Tensile properties (stress measured at the break point σ_b_, stress at yield (σ_y_) and elongation at break ε_b_) of miniextruded blends.

Blends	σ_y_ (MPa)	σ_b_ (Mpa)	ε_b_ (%)
**F1**	-	31.8 ± 1.4	572.7 ± 20.7
**F2**	10.2 ± 0.7	33.0 ± 1.2	554.2 ± 12.3
**F3**	23.3 ± 1.9	32.5 ± 1.6	543.7 ± 29.8

**Table 6:** Results of differential scanning calorimetry (DSC) analysis (first heating).

Blends	T_g_ (°C)	T_C_ (°C)	ΔH_C_ (J/g)	T_m_ (°C)	ΔH_m_ (J/g)	X_C_%
F1	43.64	96.4	3.051	143.7	18.84	27%
F2	31.77	86.71	12.97	145.3	19.94	12%
F3	30.32	85.38	13.30	144.77	19.19	11%

**Table 7:** Results of DSC analysis (second heating).

Blends	T_g_ (°C)	T_m, PBS_ (°C)	ΔH_m, PBS_ (J/g)	T_C, PLA_ (°C)	ΔH_C, PLA_ (J/g)	T_m, PLA_ (°C)	ΔH_m, PLA_ (J/g)	X_C_%
F1	26.96	79.63	3.74	88.21	11.49	144.27	22.62	19%
F2	33.54	83.40	6.83	96.72	16.90	145.40	20.54	6%
F3	31.89	83.69	7.86	95.05	15.02	144.99	20.97	11%

**Table 8:** Comparison in mechanical properties of PLA, F3 and low density polyethylene (LDPE) extruded films.

	Tensile Test on Dumbell Specimens					
	Orientation	E (GPa)	σ_y_ (MPa)	ε_y_ (%)	σ_b_ (MPa)	ε_b_ (%)
PLA pure	MD	3.31	37.6 ± 3.2	3.2 ± 0.1	30.5 ± 2.5	17.7 ± 9.8
	CD	2.60	31.8 ± 3.9	2.9 ± 0.2	26.5 ± 3.6	14.0 ± 6.8
F3	MD	0.09	2.3 ± 1.1	3.6 ± 0.9	23.3 ± 5.0	493.4 ± 25.3
	CD	0.10	2.5 ± 1.0	3.2 ± 0.5	21.9 ± 3.5	482.1 ± 41.1
LDPE	MD	0.13	6.0 ± 0.9	8.1 ± 2.3	17.7 ± 4.1	421 ± 119
	CD	0.13	3.2 ± 2.2	4 ± 0.7	10. 9 ± 1.8	685 ± 290

**Table 9:** Results of the trouser tear tests.

Blends	Critical Fracture Energy (N/m)	
	MD	CD
PLA	10,000 ± 2000	11,000 ± 1000
F3	23,000 ± 8000	29,000 ± 7000
LDPE	85,000 ± 15,000	111,000 ± 5000
